# Cytomegalovirus (CMV)-Driven Transformation: An Uncommon Cause of a Central Nervous System Pseudotumor

**DOI:** 10.7759/cureus.44188

**Published:** 2023-08-27

**Authors:** María Fernanda Tejada-Pineda, Irma Hoyo-Ulloa, Luis Alberto Ortega-Porcayo, Jesús J Baquera-Heredia, José Pablo Zárate-García, Samuel Romano-Feinholz, Juan Antonio Ponce-Gómez, Sergio M Jiménez

**Affiliations:** 1 Neurological Surgery, ABC Medical Center, Mexico City, MEX; 2 Infectious Disease, ABC Medical Center, Mexico City, MEX; 3 Pathology, ABC Medical Center, Mexico City, MEX; 4 Neurological Surgery, Instituto Nacional de Neurología y Neurocirugía "Manuel Velasco Suarez", Mexico City, MEX; 5 Radiosurgery Unit, Instituto Nacional de Neurología y Neurocirugía "Manuel Velasco Suarez", Mexico City, MEX; 6 Neurological Surgery - Radiosurgery, ABC Medical Center, Mexico City, MEX

**Keywords:** ring-enhancing lesion, cmv neurologic disease, intracranial mass lesion, cmv, hiv/aids

## Abstract

Cytomegalovirus (CMV) is an opportunistic virus that can cause life-threatening neurological diseases in immunocompromised individuals, particularly those with HIV/AIDS. In this case report, a patient presenting with left gait lateralization was found to have a ring-enhancing cerebral mass lesion that was attributed to CMV. To date, only eight similar cases have been documented. When evaluating patients with HIV/AIDS who have cerebral mass lesions, clinicians should keep CMV as a possible cause because prompt antiviral therapy may improve clinical outcomes.

## Introduction

Cytomegalovirus (CMV) neurologic disease occurs mainly in severely immunocompromised patients such as transplant recipients and patients with acquired immunodeficiency syndrome (AIDS). Neurological disease may develop especially in those who have a CD4+ T-lymphocyte count <50 cells/microL. CMV can affect the brain or the spinal cord. The most common clinical presentations are encephalitis, ventriculoencephalitis, myelitis, polyradiculopathy, and mononeuritis multiplex. A few cases of cerebral mass lesions mimicking a brain tumor have been reported [1,2].

The differential diagnosis of intracranial mass lesions in patients with AIDS includes infectious diseases such as toxoplasmosis, cryptococcosis, tuberculosis, histoplasmosis, and malignancies like central nervous system lymphoma [3]. However, viral mass lesions have rarely been reported. In this case, we describe an intracranial ring-enhancing lesion in a patient with clinical stage 4 HIV (according to the WHO HIV/AIDS Clinical Staging System) [4] in whom other diagnoses were excluded and brain histopathological studies and immunohistochemistry (IHC) reported CMV disease.

## Case presentation

A 47-year-old man was brought to the emergency room due to a nonproductive cough, somnolence, and generalized weakness that had been present during the previous two weeks but had worsened in the last four days. During the patient interview, as we reviewed different systems, he complained of diarrhea and dysphagia for the past three weeks and weight loss of 20 kg (44 lbs) in six months. On examination, he was febrile (38.2 C), and pulse oximetry on room air showed an oxygen saturation of 86%. He received oxygen through a nasal cannula at 3 liters per minute, with which normal saturation was achieved. His physical examination showed right cervical lymphadenopathies, a reddish tongue with oral candida, and a decreased vesicular murmur in both lung bases. The neurological examination was normal, except for a slight left gait lateralization.

The initial workup reported an HIV infection, with a CD4+ T lymphocyte count of 22 cells and a viral load of 798, 000 copies/mL. A gastrointestinal (GI) polymerase chain reaction (PCR) panel revealed an infection by Giardia lamblia. IgG antibodies for Toxoplasma gondii and CMV were negative, as well as hepatitis B and C serologies. QuantiFERON-TB was negative. Galactomannan and Histoplasma antigen tests were also negative.

**Figure 1:**
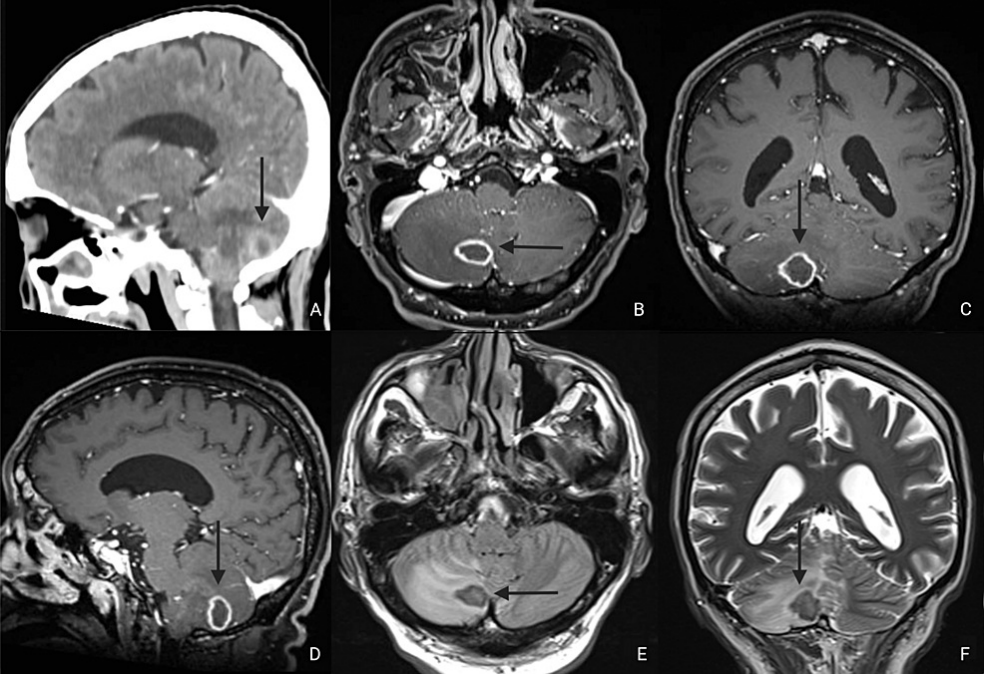
CT (A) and MRI (B-F) A: Sagittal CT with contrast showing a ring-enhancing lesion at the suboccipital cerebellar surface. B, C, D: Axial, coronal, and sagittal T1-weighted contrast imaging demonstrates an ovoid ring-enhancing lesion with a thickened subcapsular appearance in the right cerebellar hemisphere. E: Axial FLAIR showing surrounding cerebellar edema. F: Coronal T2-weighted image shows a hypointense cerebellar paravermian lesion. FLAIR: fluid-attenuated inversion recovery

Chest computed tomography (CT) showed multifocal pneumonia, as well as mediastinal and axillary lymphadenopathy. Brain CT identified an ovoid ring-enhancing lesion of 17 x 15 mm, with associated edema in the right cerebellar hemisphere that compressed the fourth ventricle (Figure 1A). Brain magnetic resonance imaging (MRI) with intravenous contrast was notable for a ring-enhancing nodular lesion in the right cerebellar hemisphere in T1 and T2 sequences, which did not show signal intensity restriction (Figures 1B-1F). Subsequently, a lumbar puncture was performed and cerebrospinal fluid (CSF) showed no cytochemical abnormalities. CSF meningitis PCR panel and cultures were negative. He was started on ceftriaxone, azithromycin, fluconazole, metronidazole, and trimethoprim-sulfamethoxazole.

Respiratory and GI symptoms improved during the following days. The brain CT was repeated after two weeks of treatment with similar results.

Surgical resection of the lesion through a suboccipital craniotomy was performed. Antiretroviral treatment was started (bictegravir, emtricitabine, and tenofovir alafenamide).

While the final brain histopathological results were pending, the patient was discharged, but he developed fever and bicytopenia during the first 24 hours. He was readmitted to the hospital for an extensive workup. A brain MRI showed complete resection of the cerebellar lesion and associated focal dural enhancement of the surgical site. The CSF-PCR was positive for CMV. The chest CT showed new pulmonary infiltrates, so a bronchoscopy was performed. The broncho-alveolar lavage had a positive PCR test for Pneumocystis jirovecci. Bacterial, fungal, and mycobacterial cultures were negative, as well as the Mycobacterium avium PCR test. Bacterial, mycobacterial, and fungal cultures taken from a bone marrow aspirate were negative.

**Figure 2:**
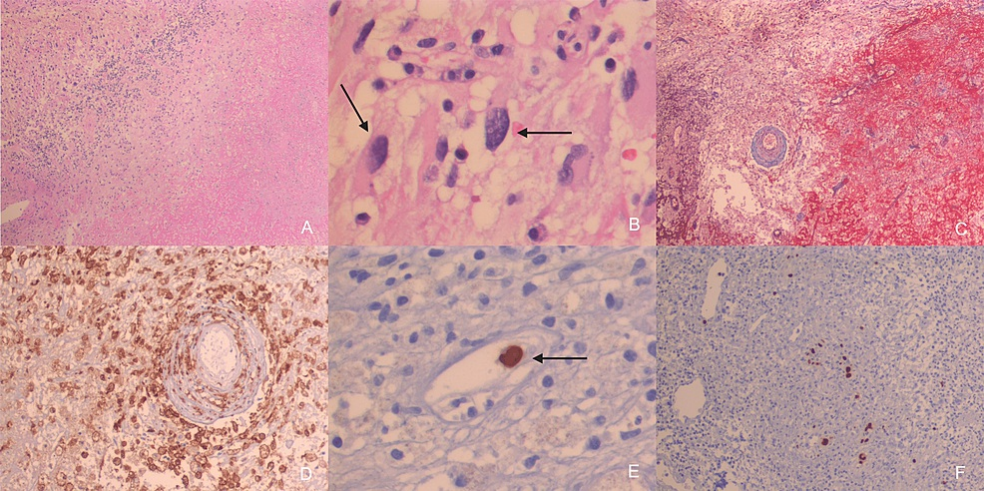
Histological behavior of CMV infection A: Brain tissue near necrotic focus shows mononuclear inflammatory cells. B: Higher magnification shows intranuclear Cowdry type A-like inclusion bodies. C: Masson's trichrome stain highlights the necrotic areas from surrounding brain tissue. D: CD163 immunohistochemistry shows dense infiltrating macrophages, suggesting a demyelinating component of the tissue response. E: Focal immunoreactivity for CMV in endothelial cells. F: Lung biopsy showing multiple infected cells.

Due to worsening diarrhea despite metronidazole, a colonoscopy was performed. It reported ulcers encircled by an erythematous halo along the entire length of the colon. CMV colitis was diagnosed through positive immunochemistry and colonic macroscopic ulcers. The brain pathology report revealed a necrotic nodule with a xanthomatous reaction and reactive gliosis with atypia, associated with the cytopathic effect of CMV. No neoplastic cells or specific microorganisms were identified (Figures 2A-2F).

The patient was treated with intravenous ganciclovir 250 mg every 12 hours for six weeks. Lung infiltrates were concluded to be immune reconstitution syndrome. During an eight-month follow-up visit, the patient's CD4+ T lymphocyte count had risen, he had an undetectable viral load, recovered weight, and was clinically well. The patient remains asymptomatic after one year.

## Discussion

There are eight reported cases of brain mass lesions caused by CMV worldwide, of which seven died shortly after diagnosis [5]. Out of those eight patients, seven were diagnosed through a brain biopsy and one through a PCR test on CSF. Two patients were treated with intravenous ganciclovir 5 mg/kg every 12 hours, one with intravenous foscarnet 100 mg/kg every 12 hours, two received ganciclovir and then foscarnet, one with foscarnet followed by ganciclovir, and the two remaining patients did not receive antiviral treatment [5,6]. Our patient had a positive CMV CSF-PCR test, brain immunohistochemistry (IHC), and cytopathic changes. He received IV ganciclovir (Table 1).

**Table 1:** Summary of reported cases of intracranial mass lesions due to CMV infection in patients with AIDS NA: not available; M: male; F: female; CT: computed tomography; MRI: magnetic resonance imaging; CSF: cerebrospinal fluid; PCR: protein chain reaction

Case report	Year	Age	Sex	CD4+ cells/mm^3^	Clinical characteristics	Imaging results (CT/MRI)	Diagnosis	Treatment	Outcome
1 [6]	1995	32	M	81	Fever, seizures, bilateral limb weakness, hyperreflexia	Frontal contrast-enhancing mass lesion	Brain biopsy	Ganciclovir	Died after 3 months of diagnosis
2 [6]	1995	31	M	14	Fever, seizures, lower limb weakness, confusion, urinary incontinence	Hemorrhagic lesion in left frontal lobe	CSF-PCR test	Ganciclovir and then foscarnet	Died after 5 months of onset of symptoms
3 [7]	1996	39	M	10	Fever, headache, left hemiparesis, frontal lobe syndrome	Ring-enhancing lesion of the right caudate nucleus and left frontal lobe	Brain biopsy	Foscarnet	Died after 10 weeks of onset of symptoms
4 [7]	1996	34	M	9	Headache, right hemiplegia, aphasia	Left ring-enhanced temporoparietal lesion	Brain biopsy	Foscarnet and then ganciclovir	Died after 6 months of onset of symptoms
5 [8]	1997	35	M	20	Left central facial palsy, left hemiparesis, psychomotor slowing	Heterogeneous enhancing lesion in right basal nuclei	Brain biopsy	Ganciclovir and then foscarnet	Died after 4 months of diagnosis
6 [8]	1997	51	F	NA	Headache, right hemiparesis, dizziness, right dysmetria, ataxic gait	Enhancing lesion in the right cerebellar hemisphere	Autopsy	Without treatment	Died after 4 weeks of admission
7 [9]	1997	31	M	10	Headache, right hemiparesis, aphasia, hyperreflexia	Low signal area in T1 weighted images in the left parietal lobe	Brain biopsy	Ganciclovir	Alive and symptom-free after 4 months of discharge
8 [5]	2003	39	F	8	Fever, seizures, psychomotor slowing	Heterogeneous enhancing lesion in left frontal lobe	CSF-PCR test	Without treatment	Died after 4 weeks of admission
Our patient	2023	47	M	22	Fever, left gait lateralization, somnolence, and generalized weakness	Ovoid ring-enhancing lesion in the right cerebellar hemisphere	CSF-PCR test and brain biopsy	Surgical resection of lesion and ganciclovir	Alive and symptom-free after 12 months of discharge

CMV infection occurs in patients with a severely low count of CD4+ T-lymphocytes (less than 50 cells/microL). According to Vidal et al., the mean count of CD4+ T-lymphocytes in patients with an intracranial mass lesion caused by CMV was 22 cells/microL, with a range between 8 and 81 cells/microL [5]. This is also relevant to our case, as our patient had advanced HIV with less than 50 CD4+ cells.

The diagnosis of CMV disease is usually made based on clinical presentation and, when feasible, the presence of the virus in the tissue. CMV antigen blood assays, blood cultures, and PCR are not recommended to diagnose end-organ disease in advanced HIV patients because they have a low positive predictive value [10]. The CSF-PCR test has a 95% sensitivity and 85% specificity to diagnose CMV neurological infection, however, brain biopsy remains the gold standard [1,11]. A positive result of a CSF-PCR test for CMV in a patient with an intracranial mass lesion should raise suspicion that CMV may be the main cause [8]. It is worth mentioning that our patient’s first CSF analysis had a negative CMV-PCR, and it was not until his fever recurred that the virus was detected on CSF-PCR. A false-negative result in PCR panels has been associated with a low quantity of the pathogen [12].

Intracranial mass lesions caused by CMV in patients with AIDS have nonspecific characteristics in neuroimaging studies; therefore, they cannot be differentiated from other more common causes of mass lesions, such as Toxoplasma gondii, and primary central nervous system (CNS) lymphoma [8,13]. In all the cases previously studied, including ours, the mass lesions were ring-enhancing lesions with surrounding edema. This may delay appropriate treatment and worsen the prognosis. Moulignier suggested that CMV should be considered a differential diagnosis when a slowly progressive ring-enhanced intracranial mass lesion in patients with advanced HIV does not respond to anti-toxoplasma treatment [7]. Our patient was first treated for CNS toxoplasmosis, but when the follow-up brain CT did not show any changes, a biopsy was performed. It is worth mentioning that in Mexico when CNS toxoplasmosis is suspected, TMP-SMX is used because sulfadiazine is no longer distributed in the country.

Data on treatment for well-documented CMV neurological disease is scarce. Dual therapy with intravenous ganciclovir (5 mg/kg every 12 hours) and foscarnet (90 mg/kg per day) is used to treat severe neurologic diseases, such as CMV encephalitis, despite substantial toxicities associated with that approach. We favored the use of ganciclovir since it is a well-studied and commonly prescribed anti-CMV drug. The role of valganciclovir has not been established.

CMV mass lesions in HIV patients have a high mortality. From the previous eight reported cases, only one patient survived. He was treated with induction doses of ganciclovir for an unknown period and received maintenance therapy for four more months [9]. Our case is the second case of successful treatment of a CMV brain lesion, in which initially surgical resection was essential for the diagnosis and induction treatment. There is no evidence regarding the extent of surgical resection in end-organ CMV disease, however, functional neuroanatomical location should be considered to minimize neurological deficit secondary to the surgery. Ganciclovir was started early, and the patient had mild neurological symptoms at the onset. Prompt invasive diagnosis, repeating CMV tests, and early treatment could have influenced the good outcome. An important take-home point should be that CMV should be considered an uncommon differential diagnosis of ring enhancement lesions in HIV patients; surgical biopsy or resection is crucial for a prompt diagnosis and effective antiviral curative treatment.

## Conclusions

Intracranial mass lesions caused by CMV in patients with AIDS are uncommon and difficult to diagnose due to nonspecific clinical and neuroimaging findings. Despite initial negative testing, a brain biopsy confirmed CMV as the underlying cause of the patient’s intracranial ring-enhancing lesion, leading to successful treatment with intravenous ganciclovir. These findings emphasize the importance of considering CMV in the differential diagnosis of mass lesions in HIV patients with a severely low CD4+ T-lymphocyte count who do not respond to toxoplasmosis treatment and have a negative CSF-PCR test. A prompt brain biopsy should be pursued in such cases. Though few comparable cases exist, our approach offers insights into management strategies. Long-term follow-up is needed due to the rarity of the condition, offering a deeper understanding of recurrence and progression-free survival.

## References

[1] Bowen LN, Smith B, Reich D, Quezado M, Nath A (2016). HIV-associated opportunistic CNS infections: pathophysiology, diagnosis and treatment. Nat Rev Neurol.

[2] Jacobson MA (2023). AIDS-related cytomegalovirus neurologic disease. UpToDate.

[3] Elicer I (2020). Approach to an intracranial mass in patients with HIV. Curr Neurol Neurosci Rep.

[4] (2023). World Health Organization. WHO case definitions of HIV for surveillance and revised clinical staging and immunological classification of HIV-related disease in adults and children. https://apps.who.int/iris/bitstream/handle/10665/43699/9789241595629_eng.pdf.

[5] Vidal JE, Dauar RF, Penalva de Oliveira AC, Coelho JF, Lins DL (2003). Cerebral mass lesion due to cytomegalovirus in a patient with AIDS: case report and literature review. Rev Inst Med Trop Sao Paulo.

[6] Dyer J, French M, Mallal S (1995). Cerebral mass lesions due to cytomegalovirus in patients with AIDS: report of two cases. J Infect.

[7] Moulignier A, Mikol J, Gonzalez-Canali G (1996). AIDS-associated cytomegalovirus infection mimicking central nervous system tumors: a diagnostic challenge. Clin Infect Dis.

[8] Huang PP, McMeeking AA, Stempien MJ, Zagzag D (1997). Cytomegalovirus disease presenting as a focal brain mass: report of two cases. Neurosurgery.

[9] Bassil HF, William DC (1997). Cytomegalovirus encephalitis in an HIV positive patient presenting with a cerebral mass lesion. AIDS Patient Care STDS.

[10] (2023). Panel on opportunistic infections in adults and adolescents with HIV: guidelines for the prevention and treatment of opportunistic infections in adults and adolescents. https://www.idsociety.org/contentassets/7ab3d1e72a9d4868be6d6c2c2553818e/adult_oi.pdf.

[11] d'Arminio Monforte A, Cinque P, Vago L (1997). A comparison of brain biopsy and CSF-PCR in the diagnosis of CNS lesions in AIDS patients. J Neurol.

[12] Vila J, Bosch J, Muñoz-Almagro C (2020). Molecular diagnosis of the central nervous system (CNS) infections. Enferm Infecc Microbiol Clin (Engl Ed).

[13] Thurnher MM, Donovan Post MJ (2008). Neuroimaging in the brain in HIV-1-infected patients. Neuroimaging Clin N Am.

